# The CXCL8-CXCR1/2 Axis as a Therapeutic Target in Breast Cancer Stem-Like Cells

**DOI:** 10.3389/fonc.2019.00040

**Published:** 2019-02-06

**Authors:** Pier Adelchi Ruffini

**Affiliations:** Research and Development Department, Dompé farmaceutici S.p.A., Milan, Italy

**Keywords:** CXCR1, CXCL8, cancer stem-like cells, reparixin, breast cancer

## Abstract

Cancer stem-like cells (CSC) have been targeted by different strategies over the last decade. This mini review focuses on preclinical and clinical results obtained by interfering with chemokine receptors CXCR1 and CXCR2 in breast cancer. This strategy is currently being tested in a randomized, double blind phase 2 clinical trial.

**Cancer stem-like cells** (CSC) have been the focus of several clinical investigations testing different strategies for a more effective anticancer treatment through inhibition of this unique cell population (1). Targeting the CXCL8-CXCR1/2 axis is one such strategy that has moved from preclinical models to an ongoing randomized phase 2 clinical trial in breast cancer.

**CXCL8** (formerly IL-8) is a chemokine whose biological effects are mediated by two G-protein-coupled receptors: **CXCR1** and **CXCR2** (2). CXCL8 has been reported to play multiple roles in cancer, such as increasing proliferation, angiogenesis, invasion, and metastases (3). In **breast cancer**, recent evidence points to this chemokine as a key regulator of CSC activity (4).

## Preclinical Evidence in Breast Cancer

In breast cancer, tumor cells capable of forming tumors in immunocompromised mice (i.e., CSC by a functional definition) are identified by the expression of either the enzyme aldehyde dehydrogenase (ALDH) (5) and/or the CD24^−^/CD44^+^ phenotype (6), representing two largely non-overlapping cell populations. CXCR1 was identified as a druggable target on breast cancer CSC identified by the expression of ALDH, while its expression was almost undetectable on bulk (i.e., non-CSC) tumor cells (7). In keeping, breast cancer CSC were shown to proliferate *in vitro* in response to the addition of exogenous CXCL8 while a small molecular weight antagonist of CXCR1/2 (**reparixin**) (8) or a blocking anti-CXCR1 (but not anti-CXCR2) monoclonal antibody were both able to deplete CSC *in vitro* (9). A FAS-FASL mediated bystander effect killed the vast majority of bulk tumor cells *in vitro*, suggesting the possibility of synergistic effects with chemotherapy (9). In human breast cancer cell lines or breast cancer patient-derived xenografts orthotopically implanted in mice, the combination of weekly docetaxel and reparixin for 4 weeks was more effective than either treatment alone in reducing tumor size (9). However, in tumors recovered from mice that had been treated with reparixin, either alone or in combination with chemotherapy, CSC proportion was far lower than in tumors recovered from mice receiving chemotherapy alone (9). These results were framed in a model where, following administration of chemotherapy, CXCL8 and FASL are released by dying bulk tumor cells. Engagement of CXCR1 on the surface of CSC by CXCL8 shelters CSC from apoptotic signals delivered by FASL. To the contrary, when CXCR1 signaling on CSC is blocked by reparixin these cells undergo FASL-mediated apoptosis. Evidence provided later by independent laboratories supports this model. First, as originally reported by Ginestier and coworkers, tumor cells exposed to taxane *in vitro* release CXCL8 (10). Also, Triple-Negative Breast Cancer (TNBC) tumor cells recovered from immunocompromised mice following two doses of paclitaxel displayed a marked and dose-dependent increase in mammosphere forming efficiency as compared with untreated mice (10). Furthermore, and again in line with the original report by Ginestier, administration of a CXCR1 inhibitor reduced CSC percentage *in vitro*. Consistent findings were later reported by an independent group (11). Second, in breast cancer patients with pleural effusions and/or ascites CXCL8 levels were measured and tumor cells recovered and cultured *in vitro* (4). A direct correlation was observed between CXCL8 levels and CSC activity by means of mammosphere formation (4). Surface CXCR1 was detected on the majority of mammosphere cells, and the effects of exogenous CXCL8 on mammosphere formation were blocked by a CXCR1/2 inhibitor, SCH563705 (4).

The relative contribution of CXCR1 inhibition and paclitaxel in this model were further investigated in CSC-enriched mammospheres from the human TNBC cell line MDA-MB231. The combination treatment displayed a synergistic effect on mammosphere number and an additive effect on mammosphere volume as compared with either treatment alone (12). Different than paclitaxel, which increased the number of dead cells, reparixin increased the number of non-proliferating cells, and the combination treatment exerted both effects (12). In keeping with previous reports (9), also in MDA-MB231-derived tumorspheres reparixin activity was mediated by inhibition of the FAK/AKT pathway which is unaffected by paclitaxel. When the effects on cell cycle were investigated, a shift of tumor cells in S phase or a block in G2 phase were observed upon paclitaxel and combination treatment, respectively. In keeping, cyclin B1, which is responsible for the cell cycle progression from G2 to S phase, was also inhibited by the combination treatment (12). Furthermore, paclitaxel + reparixin treatment induced “cell senescence by decreasing PI3K-Akt activation paralleled by a decrease of the cytosolic p-FOXO3A (inactive) and by an increase of p27” (12). The effects on cell cycle, cyclin B1 and p-FAK levels recorded upon exposure to reparixin were reproduced using neutralizing anti-CXCR1 and anti-CXCL8 monoclonal antibodies, thus providing indirect evidence of the ability of reparixin to downregulate CXCL8-CXCR1signaling pathway (12).

Another set of experiments aimed at testing the hypothesis that inhibition of CSC would reduce metastatic spread. First, it was shown that reparixin administration reduced metastasis formation in mice following injection of luciferase-transfected human breast cancer cells into the bloodstream (9). Second, the suppressive activity of CXCR1 inhibition on the metastatic process was tested in a mouse model of brain metastases by the TNBC cell line MDA-MB231. In the absence of brain metastases, reparixin does not cross the blood brain barrier (BBB). However, in the presence of brain metastases and an allegedly damaged BBB, reparixin can be found in the central nervous system (12). When treatment was started on the same day when tumor cells were injected, a significant decrease of both the number and the volume of brain metastases was observed following single agent (i.e., reparixin or paclitaxel) as well as the combination treatment. When treatment was started at day 7 following tumor cell injection and continued until day 21, a significant reduction of the number of brain metastases was observed only following combination treatment, which also showed a trend toward an inhibitory effect on metastases volume (12).

## Preclinical Evidence in Tumors Other Than Breast Cancer

Anti-tumor and anti CSC activity of reparixin has been demonstrated in human epithelial thyroid cancer *in vitro* and *in vivo* (13). Reparixin ability to inhibit stemness (evaluated by stemness marker expression and tumorsphere formation) and epithelial-mesenchymal transition (EMT) (evaluated at both the biochemical and functional level) of thyroid cancer was shown to be dependent, different than in breast cancer (9), on its activity on both CXCR1 and CXCR2 (13).

In malignant melanoma, CXCR1/2 inhibition reduced the percentage of ALDH+ cells in human tumors growing in nude athymic mice (14).

In pancreatic cancer (15) a positive correlation was found between CXCR1 and both CD44 and CD133 stemness marker expression. Exogenous CXCL8 added to pancreatic cancer cells *in vitro* increased their invasion ability, tumorsphere formation, and CSC population and addition of a CXCR1-blocking monoclonal antibody was able to revert all these effects (15).

## Clinical Trials in Breast Cancer

In a phase Ib study (NCT02001974) (16), patients with HER-2 negative metastatic breast cancer not known to be refractory to paclitaxel who had received no more than three lines of cytotoxic chemotherapy in the metastatic setting were enrolled in cohorts of 3–6 patients to receive escalating doses of the CXCR1/2 inhibitor reparixin oral tablets three times per day (t.i.d.) from day 1 to 21 in combination with a fixed dose of weekly paclitaxel (80 mg/m^2^) on days 1, 8, and 15 of a 28-days cycle, for as long as clinical benefit was observed. Primary objectives were the assessment of the safety of the combination and the pharmacokinetic (PK) profile of oral reparixin. Expansion of the highest dose cohort was foreseen to gain additional PK and safety data. Cohorts 1–3 received reparixin 400, 800, and 1,200 mg t.i.d. respectively. In cycle 1 only, patients received a 3 days course of reparixin alone (day −3 to −1) at the assigned dose for the cohort, for purpose of obtaining single agent PK data.

Thirty-three patients were enrolled in the study. Eighty-three percent of patients had visceral disease, and the majority had two or more sites of metastasis. 20/33 patients had received prior (neo)adjuvant chemotherapy, and 16 of these patients had received a taxane in the (neo)adjuvant setting. 19/33 had received chemotherapy in the metastatic setting, with 11 having one prior metastatic regimen and eight having two or more chemotherapy regimens. Thirty patients were evaluated for safety. There were no dose limiting toxicities in any cohort. Most adverse reactions (ADR) were of grade 1 (79.8%), with only 2.7% grade 3 ADR. There was no apparent dose effect of increasing reparixin dose on the incidence, severity or profile of treatment emergent adverse events (TEAE) experienced by the treatment groups, and there were no clinically significant differences between the treatment groups with regards to laboratory measurements, vital signs, ECG, and physical examination assessments. Twenty-seven patients were evaluated for antitumor activity. In total, 8/27 patients had a confirmed RECIST response. Of responding patients, all but one were from cohort 3. Median time to progression (TTP) (95% C.I.) for the 3 cohorts were 58 days (44-infinity) for cohort 1, 67 days (58–82) for cohort 2 and 162 days (60–229) for cohort 3. Remarkably, there were long term remissions among patients treated (16). In this trial, it was not possible to obtain optional serial biopsies of tumor tissue at study entry and during treatment from any patient. However, blood-based biomarkers of CSC were explored. The circulating biomarkers included Circulating Tumor Cell (CTC) enumeration, evaluation of ALDEFLUOR, and EMT transcription factors in peripheral blood, and serum cytokine measurements. Unfortunately, no clear pattern of change in any of these markers was observed. This is likely related to multiple issues, including but not limited to small sample size, low CTC number in the enrolled patient population leading to limited tumor material for testing, and high baseline heterogeneity in the measurements.

Operable breast cancer is a more suitable clinical setting to evaluate the ability of a novel agent to reduce the number of CSC following treatment, as they can be measured on readily available tumor tissue. Thus, after reviewing safety data from the second cohort of the above trial, a window-of-opportunity, pilot trial (NCT01861054) of single agent reparixin was started (17).

Patients with previously untreated HER-2 negative operable breast cancer not eligible for neo(adjuvant) treatment were divided into two cohorts, i.e., group A: histologically proven ER^+^ and/or PgR^+^ and group B: ER^−^/PgR^−^ breast cancer (i.e., TNBC). This design allowed potential to identify the cohort of patients who might benefit the most from this treatment in later stage clinical trials. Oral reparixin was administered at 1,000 mg t.i.d. for 21 consecutive days before curative surgery. Core biopsies were taken at baseline (day −14 to 0) and at the completion of therapy (day 21). The primary objectives of this study were to evaluate the effects of orally administered reparixin on CSC in the primary tumor and the tumor microenvironment and to evaluate the safety of oral reparixin. Signal of activity was defined as a ≥20% reduction of CSC (defined by either the ALDH^+^ or CD24^−^/CD44^+^ phenotype) in tumor tissue from baseline values as measured by flow cytometry accompanied by a consistent reduction of the same cell population by immunohistochemistry (IHC).

A total of 20 patients were enrolled, 18 of whom in group A. Signal of activity was detected by flow cytometry in the majority of patients (18), but the very low numbers of CSC hindered the possibility to confirm flow cytometry results by IHC. However, the later published evidence that the two breast cancer CSC populations (i.e., ALDH^+^ and CD24^−^/CD44^+^) investigated reside in different areas of primary breast tumors and can transition from one phenotype to the other (19) might affect the reliability of CSC counts in this patient population. More in general, the clinical relevance of a ≥20% reduction of CSC following a single 21-day course of reparixin in this patient population is unknown and was beyond the scope of this trial.

From a safety standpoint, also in this trial reparixin appeared to be well-tolerated with 10/20 patients experiencing one or more ADR, all of which of grade ≤ 2. Neither TEAE leading to treatment discontinuation nor delays in surgery due to TEAE were recorded.

## Conclusions

Evidence for a CXCL8-CXCR1/2 axis in CSC has been reported by independent laboratories and offers a potential therapeutic target. Clinical trials aimed at testing the effective targeting of CSC through this axis have been conducted in breast cancer, where the most information is available from preclinical research. Reparixin appeared to be well-tolerated, however, such trials were faced with several issues for efficacy evaluation, e.g., the very low numbers of CSC in primary operable breast cancer. To circumvent this limitation, circulating markers for monitoring the effect of anti-CSC agents were explored but these assays turned out to be inadequate.

Future prospects for CSC targeting agents include the development of reliable assays to measure stem cell number and/or activity (20) in serial biopsies from accessible tumors (e.g., window-of-opportunity trials), and alternative endpoints in clinical trials in the metastatic setting. One possible endpoint is the development of metastases at new sites (21), which can have also clinical significance (22). In keeping with preclinical findings (9, 12), it is hypothesized that an effective anti-CSC treatment will impact on development of new metastases while progression of pre-existing metastases is more consistent with proliferation of non-CSC, bulk tumor cells that should be addressed by chemotherapy.

As concerns CXCR1/2 inhibition, a randomized, placebo-controlled clinical trial (NCT02370238) of weekly paclitaxel with and without reparixin in front line treatment of metastatic TNBC has completed enrolment. Identification of clinical (e.g., disease sensitivity to chemotherapy) and/or cellular/molecular biomarkers of patients most likely to benefit from treatment represents a future direction of research, while analysis of time to new metastasis may fuel development of this strategy in the (neo)adjuvant setting, also leveraging on safety data generated in metastatic patients.

## Author Contributions

The author confirms being the sole contributor of this work and has approved it for publication.

### Conflict of Interest Statement

PAR is a full time employee of Dompé farmaceutici S.p.A.
